# The association of glycemic index and glycemic load with elevated blood pressure in Iranian women

**DOI:** 10.15171/jcvtr.2019.45

**Published:** 2019-10-24

**Authors:** Seyedeh Forough Sajjadi, Alireza Milajerdi, Leila Azadbakht

**Affiliations:** ^1^Department of Community Nutrition, School of Nutritional Sciences and Dietetics, Tehran University of Medical Sciences, Tehran, Iran; ^2^Diabetes Research Center, Endocrinology and Metabolism Clinical Sciences Institute, Tehran University of Medical Sciences, Tehran, Iran; ^3^Food Security Research Center and Department of Community Nutrition, School of Nutrition and Food Science, Isfahan University of Medical Sciences, Isfahan, Iran

**Keywords:** Elevated Blood Pressure, Glycemic Index, Glycemic Load, Obesity

## Abstract

***Introduction:*** Dietary intake is a risk factor related to elevated blood pressure (EBP). Few studies have investigated an association of dietary glycemic index (GI) and glycemic load (GL) with the EBP. The aim of the current study was to examine the association of dietary GI and GL with the EBP among a group of healthy women.

***Methods:*** This population-based cross-sectional study was conducted on 306 healthy women. Dietary GI and GL were measured using a validated semi-quantitative food frequency questionnaire (FFQ). Blood pressure (BP) was measured twice by a mercury sphygmomanometer from the right arm. Anthropometric measurements were also assessed according to the standard protocols.

***Results:*** Before controlling for potential confounders, no significant association was seen between dietary GI/GL and SBP/DBP. Also after controlling for potential confounders, the associations did not change between dietary GI and SBP (odds ratio [OR]: 0.96; 95% CI: 0.42-2.17, *P* = 0.87), between GI and DBP (OR: 0.72; 95% CI: 0.35-1.45, *P* = 0.37), as well as between GL and SBP (OR: 1.04; 95% CI: 0.43-2.49, *P* = 1.00) and between GL and DBP (OR: 1.20; 95% CI: 0.56-2.00, *P* = 0.61). In a stratiﬁed analysis by obesity and overweight, differences between tertiles of GI were not significant (OR: 0.75; 95% CI: 0.42-1.31, *P* = 0.31), even after adjustment for the potential confounders (OR: 1.54; 95% CI: 0.70-3.40, *P* = 0.26).

***Conclusion:*** This study did not show a significant association between dietary GI/GL and the risk of high SBP/DBP. In addition, no significant association was found between dietary GI/GL and odds of overweight or obesity in adult women.

## Introduction

Elevated blood pressure (EBP), based on adult treatment panel III, is defined as a systolic blood pressure (SBP) ≥130 mmHg and/or diastolic blood pressure (DBP) ≥85 mm Hg.^1^ Prevalence of hypertension has gently increased in the 2 last decades. Hypertension has been estimated to cause annually 7.5 million deaths worldwide.^2,3^ Unfortunately, prevalence of hypertension is high among Iranians.^4,5^ Hypertension causes different micro- and macro- vascular complications and increases all-cause and cardiovascular disease mortality.^6^ As the high complications due to EBP, its prevention is a public health priority, worldwide.

Several risk factors including genetic, environmental, lifestyle and psychosocial factors as well as dietary intake have been linked to the risk of EBP.^7,8^ Among them, dietary intake is one of the main environmental risk factors related to EBP.^8,9^ For instance, intakes of sodium, potassium, calcium, magnesium, iron, phosphorus or a combination of these modalities lower blood pressure (BP).^9-12^ In addition, dietary intake of carbohydrates has also found to be in relation with BP.^10,11,13^ Furthermore, some studies have found no association between dietary intake of some carbohydrates and BP and showed that increased intake of dietary carbohydrate could not elevate BP,^10^ this association was direct in some others which demonstrated diets high in carbohydrate are associated with slightly higher BP.^14^ It seems that the association strongly depends on amount and type of the ingested carbohydrates. glycemic index (GI) is an indicator of dietary carbohydrate quality, which defines as the potential of carbohydrate containing food to increase post-prandial blood glucose.^10,15^ Previous studies have shown that adherence to a low GI diet may reduce BP.^16,17^ Likewise, other studies that demonstrated excessive dietary intake of carbohydrates, particularly from high GI carbohydrates, have found to increase BP.^9,20^In contrast, some other studies reported no association between dietary GI and SBP/DBP.^19,21^

Besides to dietary GI, glycemic load (GL) is as a measure of carbohydrate quality and quantity, represents both the GI and amount of the ingested carbohydrate.^22^ In this regard, some studies have shown a significant association between consumption of a low GL diet and decreased BP,^11^ while others failed to find any association.^23,24^ It should be noted that higher post-prandial glycemic response due to consumption of high GI/GL carbohydrates causes hyperinsulinemia. Hyperinsulinemia has suggested increasing sympathetic nervous system activity, which enhance heart rate, cardiac output, vascular resistance, sodium retention and thus BP.^14^

Despite previous studies investigated the association of GI/GL with BP in western countries; few studies have investigated this association among Iranian population, especially among healthy women. High consumption of carbohydrates including white bread and white rice, which are mainly high in GI/GL,^25^ is common among Iranians. We conducted this study to investigate association of dietary GI/GL with EBP among healthy women referred to Tehran health centers.

## Materials and Methods

### Subjects and study protocol

This is a cross-sectional study on women referring to health centers affiliated to Tehran University of Medical Sciences. Based on inclusion criteria, 306 women who were referred to health centers were selected. Inclusion criteria were satisfaction to participate in the study, being Iranian, and lack of chronic diseases such as diabetes, cardiovascular disease, hypertension, cancer, liver and kidney diseases. Those who were immigrant, pregnant or lactating were excluded. Subjects with energy intake fewer than 800 or above 4200 kcal/d were also excluded. All participants signed an informed written consent before the entrance.

### Assessment of exposure

A validated and reliable 168-item food frequency questionnaire (FFQ) was used to assess dietary intake of participants. This semi-quantitative questionnaire consists of standard portion sizes for each food item and has been designed according to the Willett method. Participants were asked to determine the frequency of consumption of each food item during the previous year, based on serving sizes. Validity and reliability of the FFQ were determined previously.^26^ Food intakes reported in household measures converted to grams of food per day using the Nutritionist IV software.

Total GI of participants’ diet was estimated using the following formula: ∑ (GIa * available carbohydratea)/total available carbohydrate.^27^ Available carbohydrate of food items was calculated as total carbohydrate minus fiber. The total carbohydrates and fiber of 85 carbohydrate-containing food items were derived from the United State Department of Agriculture food-composition table. However, GI for 6 foods was derived from Iranian national tables.^28^ In addition, GI values for other 62 foods were derived from the international references.^29,30^ GI values for the rest food items which were not found neither in Iranian nor in international tables, such as some traditional desserts and sweets, were estimated based on physically and chemically similar foods.^31^ For instance, GI value of gaz, which is chiefly made of nuts and sugar (almond or pistachios), was considered to be the same as sugar. In addition, gooshfil which mainly contains sugar and white flour, was considered the same as English muffin bread. GI values of rice and dates which have different brands, were estimated as the mean values. All derived GI values were relative to glucose as the reference food. The GIs of mixed meals were calculated based on GIs of each individual food components.^27^ Dietary GL was estimated as (total GI * total available carbohydrate)/100^32^ and expressed as g/d.

### Assessment of outcome

BP was measured by a professional clinic staff using a mercury sphygmomanometer from the right arm with appropriately sized BP cuffs. BP was measured in a sitting position and underwent measurements twice. The first measurement was taken after 5 minutes of resting. After a 15-minute rest, a second measurement was taken; the average of the 2 values is reported. In this study the description of EBP, based on adult treatment panel III, used to define a high SBP (≥130 mm Hg) and/or high DBP (≥ 85 mm Hg).

### Assessment of anthropometric measures

Anthropometric measurements including participants’ weight, height and waist circumference were measured according to the standard protocols.^33^ Height and weight were measured when wearing minimal clothing and without shoes using stadiometer and digital scale to the nearest of 0.1 cm and 100 g, respectively. Body mass index (BMI) was calculated as weight (kg) divided by height squared (m^2^). Waist circumference was measured in the middle of distance between the lowest gear and top of the iliac crest (narrowest girth), in a standing position and at the end of normal exhalation, when wearing minimum clothing to the nearest of 0.1 cm.

### Assessment of other variables

General information including participants’ name, age, gender, location and contact number were recruited using a questionnaire. Socio-economic status of participants was determined by a questionnaire using family size (≤4, >4 persons), education status (academic or non-academic), and house ownership (yes, no). The score of 1 was given in case of having family members of ≤4, academic educations, and house ownership. Participants who had family members of >4, had non-academic educations, or were not house owners, were given the score of 0. The Socio-economic status score was obtained from summing up of these scores, which gave a score of 0 (poor), 1 (middle class) and 2 (high). We recorded the regular use of drugs that affect BP, including the pain medications, caffeine, and herbal supplement which used by participants then adjust the effect of drugs on BP and weight in analysis. Participants were also asked to record their daily activities during 24 hours. Mean physical activity was estimated using the following equation: PA mean = ∑ (time activity × MET). Where, PA mean is the mean physical activity, Time activity is the total time of each activity within a day, and MET is the metabolic equivalent adapted from a reference list.^34^

### Statistical analysis

All statistical analysis was performed using the IBM SPSS software version 22.0 (SPSS, Chicago, IL, USA). Normal distribution of data was checked by the Kolmogorov-Smirnov test. One-way ANOVA was used for assessed differences between tertiles of dietary GI and dietary GL. The differences between dietary intakes of participants based on the tertiles of dietary GI and dietary GL were assessed by one-way ANOVA. Logistic regression was used to assess the association of dietary GI and GL and the risk of high BP and the risk of overweight and obesity among women. Three models were constructed. Model 1 was adjusted for age and energy intake. Model 2 was adjusted for marriage status, education, occupation, number of children, family size, supplement use, medications use, and physical activity (METs/d), and Model 3 additionally adjusted for dietary intake of fiber and magnesium. Results were presented as odds ratios (ORs) and 95% con­fidence intervals (CIs) compared with the tertiles of dietary GI and dietary GL.

## Results

General characteristics of participants throughout tertiles of dietary GI and dietary GL are indicated in Table 1. Totally, data on 306 women (age 32.42 ± 8.35 years, BMI 24.64 ± 4.68 kg/m^2^) were analyzed in the current study. Participants with the highest dietary GI tended to have lower height (*P* = 0.01) and weight (*P* = 0.03) than those with the lowest dietary GI. However, differences in age (*P* = 0.36), BMI (*P* = 0.23), waist circumference (*P* = 0.20), SBP (*P* = 0.10), DBP (*P* = 0.22), physical activity (*P* = 0.16), and marriage status (*P* = 0.52) were not significant throughout tertiles of dietary GI. In addition, subjects in the highest tertile of dietary GL had lower physical activity (*P* = 0.05) as compared with those at the lowest tertile. Significant differences in education were found between tertiles of dietary GI and GL (both *P* = 0.04). However, no significant differences were found in terms of age (*P* = 0.12), weight (*P* = 0.34), height (*P* = 0.22), BMI (*P* = 0.33), waist circumference (*P* = 0.39), SBP (P=0.94), DBP (*P* = 0.35), and marriage status (*P* = 0.99) between tertiles of dietary GL.

**Table 1:** Socio-demographic characteristics of subjects according to the tertiles of dietary glycemic index and dietary glycemic load

**Variables**	**N**	**Dietary glycemic index**				**Dietary glycemic load**			
		**1**	**2**	**3**	**P**	**1**	**2**	**3**	***P*** ^ 1 ^
**<59.1**	**59.1-61.96**	**>61.96**	**<182.49**	**182.49-230.5**		**>230.5**			
Age (y)	306	32.75±8.19^2^	33.03±8.19	31.47±8.61	0.36	32.98±7.96	33.24±7.96	31.05±8.36	0.12
Weight (kg)	306	68.33±13.27	65.34±11.45	63.83±12.41	**0.03**	65.84±12.51	64.53±11.66	67.11±13.26	0.34
Height (m)	306	163.96±5.13	163.29±5.57	161.76±5.52	**0.01**	163.67±5.48	163.00±5.03	162.35±5.84	0.22
BMI (kg/m^2^)	306	25.29±5.01	24.31±4.32	24.32±4.67	0.23	24.51±4.71	24.23±4.18	25.18±5.10	0.33
Waist circumference (cm)	306	87.05±12.46	84.27±11.58	84.98±10.60	0.20	85.35±11.66	84.35±9.78	86.58±13.09	0.39
SBP (mm Hg)	306	116.72±13.28	114.12±12.91	112.82±13.55	0.10	114.90±13.48	114.32±12.62	114.45±13.90	0.94
DBP (mm Hg)	306	73.89±6.12	72.89±6.23	72.42±6.31	0.22	73.01±6.06	73.72±5.76	72.46±6.82	0.35
Physical activity (METs/d)	306	31.42±3.73	30.77±3.21	30.50±3.52	0.16	30.77±3.56	31.55±3.65	30.37±3.21	**0.05**
Marriage status (%)									0.99
Married	136	53.5%	52.5%	59.8%		55.4%	54.9%	55.4%	
Single	168	46.5%	47.5%	40.2%	0.52	44.6%	45.1%	44.6%	
Education (%)									
Low-educated	101	4.0%	17.8%	78.2%		4.0%	3.9%	10.9%	**0.04**
Diploma	102	6.9%	30.7%	62.4%		20.8%	30.4%	30.7%	
Academic	103	7.8%	33.3%	58.8%	**0.04**	75.2%	65.7%	58.4%	

BMI, body mass index; SBP, systolic blood pressure; DBP, diastolic blood pressure. ^1^*P* values are resulted from ANOVA for continues variables and chi-square test for qualitative variables. ^2^Data are indicated as mean ± SD otherwise indicated.

Dietary intakes of participants based on tertiles of dietary GI and GL are shown in Table 2. Dietary intakes of total energy (*P* = 0.006), protein (*P* = 0.001), fat (*P* = 0.0001), vitamin B2 (*P* = 0.001), vitamin C (*P* = 0.0001), β-carotene (*P* = 0.0001), calcium (*P* = 0.0001), potassium (*P* = 0.0001), fruit (*P* = 0.0001), vegetable (P=0.0001), dairy (*P* = 0.0001) and meat (*P* = 0.0001) were significantly lower among participants in the 3rdtertile than those in the first tertile of dietary GI. Furthermore, participants in the highest tertile of dietary GI had higher intakes of carbohydrate (*P* = 0.0001), vitamin B1 (*P* = 0.0001), and refined grains (*P* = 0.0001) as compared to those in the lowest tertile. With regards to the dietary GL, lower intakes of protein (*P* = 0.0001), vitamin B2 (*P* = 0.0001), calcium (*P* = 0.002), and potassium (*P* = 0.0001) were found among subjects in the 3rd versus those in the first tertile of dietary GL. In addition, participants with the highest dietary GL had significantly higher intake of energy (*P* = 0.0001), carbohydrate (*P* = 0.0001), fat (*P* = 0.002), vitamin B1 (*P* = 0.03), whole grains (*P* = 0.0001), refined grains (*P* = 0.0001) and fruits (*P* = 0.04)than those with the lowest dietary GL. Differences in intakes of vitamin C (*P* = 0.16), vitamin E (*P* = 0.21), β-carotene (*P* = 0.26), sodium (*P* = 0.20), vegetable (*P* = 0.89), dairy (*P* = 0.18) and meat (*P* = 0.90) were not statistically significant between the highest as compared to the lowest tertile of dietary GL.

**Table 2:** Dietary intakes of participants based on the tertiles of dietary glycemic index and dietary glycemic load

**Variables**	**Tertilesof dietary glycemic index**				**Tertilesof dietary glycemic load**			
	**1 (n=102)**	**2 (n=102)**	**3 (n=102)**	***P***	**1 (n=102)**	**2 (n=102)**	**3 (n=102)**	***P*** ^ 1 ^
Energy (kcal)	2289.17±704.10^2,3^	2238.69±712.98	1998.93±622.88	**0.006**	1877.81±490.70	2083.84±647.46	2565.15±727.57	**0.0001**
Protein (g)	81.58±15.85	76.78±19.51	73.59±9.40	**0.001**	82.31±20.20	77.07±12.14	72.56±12.23	**0.0001**
Carbohydrate (g)	318.9±37.67	332.99±30.03	340.91±29.16	**0.0001**	316.83±30.64	330.76±29.41	345.27±34.77	**0.0001**
Fat (g)	70.17±14.49	64.76±15.30	61.74±12.35	**0.0001**	61.06±20.05	63.07±25.84	72.52±28.18	**0.002**
Vitamin B1 (mg)	1.84±0.36	1.91±0.38	2.06±0.03	**0.0001**	1.86±0.33	1.96±0.25	1.99±0.48	**0.035**
Vitamin B2 (mg)	2.24±0.48	2.01±0.40	1.74±0.35	**0.0001**	2.13±0.44	1.98±0.44	1.87±0.47	**0.0001**
Vitamin C (mg)	163.56±69.61	132.15±55.42	108.74±46.74	**0.0001**	140.01±51.78	139.22±65.21	125.21±67.50	0.160
Vitamin E (mg)	10.67±4.90	10.59±4.65	12.08±5.91	0.071	11.53±5.25	11.43±5.25	10.38±5.10	0.217
β-Carotene (µg)	907.22±1073.33	600.68±448.41	473.01±430.92	**0.0001**	705.82±69.88	924.51±91.54	521.82±51.66	0.268
Calcium (mg)	1130.03±309.54	1002.61±269.71	867.79±181.62	**0.0001**	1076.46±297.77	977.95±257.90	946.03±267.89	**0.002**
Potassium (mg)	3730.25±833.88	3185.97±892.58	2754.78±552.33	**0.0001**	932.87±92.36	785.24±77.75	774.93±76.72	**0.0001**
Sodium (mg)	4998.15±2869.99	5215.41±2425.15	5235.11±2150.25	0.754	5431.51±2571.15	5200.24±2481.00	4816.92±2411.61	0.206
Whole grain (g)	5132.66±79.30	5131.01±86.47	5113.03±72.47	0.150	5101.16±65.83	5119.96±62.57	5155.57±97.29	**0.0001**
Refined grain (g)	281.17±108.12	373.57±104.81	481.80±137.02	**0.0001**	274.66±89.87	388.14±89.87	473.74±89.87	**0.0001**
Fruit (g)	468.12±248.43	388.25±187.66	265.49±151.22	**0.0001**	338.05±189.14	370.39±219.74	413.42±232.15	**0.043**
Vegetable (g)	466.59±241.43	338.55±157.70	291.35±160.10	**0.0001**	366.58±200.18	371.63±212.57	358.28±200.12	0.895
Dairy (g)	614.65±277.33	529.15±270.40	371.25±168.49	**0.0001**	479.06±230.35	492.28±261.26	543.70±292.47	0.180
Meat (g)	147.19±85.39	123.40±55.35	108.18±50.40	**0.0001**	125.70±66.02	124.50±65.79	128.56±70.58	0.907

^1^ *P* values are resulted from ANOVA (Analysis of variance). ^2^  Mean ± SD. ^3^ Data are adjusted for energy intake.

Multivariate-adjusted models with 95% confidence intervals for risk of high SBP and DBP across tertiles of dietary GI and GL have been indicated in Table 3. In the crude model, no significant association was found between GI with SBP and DBP (for SBP: OR: 0.71; 95% CI: 0.34-1.30, 0.30; for DBP: OR: 0.61; 95% CI: 0.34-1.10, *P* = 0.10). In addition, no significant correlation was found between GL with SBP and DBP (for SBP: OR: 1.11; 95% CI: 0.34-1.38, *P* = 0.73; For DBP: OR: 0.95; 95% CI: 0.53-1.72, *P* = 0.88). Furthermore, after adjustment for the confounders including age, energy intake, marriage status, education, occupation, number of children, family size, supplement and medications use, physical activity, as well as dietary intake of fiber and magnesium in the final model, the associations remained unchanged; Such that no significant association were found between GI and SBP (OR: 0.96; 95% CI: 0.42-2.17, *P* = 0.87), GI and DBP (OR: 0.72; 95% CI: 0.35-1.45, *P* = 0.37), GL and SBP (OR: 1.04; 95% CI: 0.43-2.49, *P* = 1.00), as well as between GL and DBP (OR: 1.20; 95% CI: 0.56-2.00, *P* = 0.61).

**Table 3:** Association of dietary glycemic index and glycemic load and the risk of high blood pressure among Tehranian women

	**Glycemic index**				**Glycemic load**			
	**1**	**2**	**3**	***P*** ^a^	**1**	**2**	**3**	***P***
High systolic blood pressure								
Crude	1	0.66 (0.34, 1.30)	0.71 (0.34,1.30)	0.30	1	0.68 (0.34, 1.38)	1.11 (0.34,1.38)	0.73
Model 1^1^	1	0.63 (0.31, 1.27)	0.86 (0.43,1.74)	0.63	1	0.59 (0.28, 1.24)	0.92 (0.43,1.99)	0.80
Model 2^2^	1	0.64 0.31, 1.32)	0.81 (0.37,1.73)	0.53	1	0.48 (0.22, 1.05)	0.86 (0.38,1.94)	0.65
Model 3^3^	1	0.70 (0.33, 1.48)	0.96 (0.42,2.17)	0.87	1	0.52 (0.24, 1.16)	1.04 (0.43,2.49)	1.00
High diastolic blood pressure								
Crude	1	0.74 (0.41, 1.31)	0.61 (0.34,1.10)	0.10	1	1.19 (0.66, 2.12)	0.95 (0.53,1.72)	0.88
Model 1	1	0.71 (0.40, 1.27)	0.61 (0.33,1.11)	0.10	1	1.19 (0.66,2.15)	1.03 (0.53, 1.97)	0.90
Model 2	1	0.75 (0.41, 1.38)	0.69 (0.36,1.33)	0.26	1	1.03 (0.55, 1.92)	1.05 (0.52,2.09)	0.88
Model 3	1	0.77 (0.41, 1.45)	0.72 (0.35,1.45)	0.37	1	1.06 (0.56, 2.00)	1.20 (0.56,2.00)	0.61

^a^ *P* values are from logistic regression. ^1^Model 1: Adjusted for age and energy intake. ^2^Model 2: Further adjusted for marriage status, education, occupation, number of children, family size, supplement use, medications use, and physical activity (METs/d). ^3^Model 3: Further adjusted for dietary intake of fiber and magnesium.

Table 4 shows multivariate-adjusted models with 95% confidence intervals for risk of obesity and overweight across tertiles of dietary GI and GL. In the crude model before adjustment for the confounders, participants in the highest tertile of GL were at 80% higher risk for obesity and overweight (OR: 1.80; 95% CI: 1.02-3.16, *P* = 0.03) than those in the lowest tertile. After controlling for the potential confounders, the association disappeared. In addition, differences in obesity and overweight between the highest rather the lowest tertile of GI were not significant (OR: 0.75; 95% CI: 0.42-1.31, *P* = 0.31). This association remained non-significant after controlling for the age, energy intake, marriage status, education, occupation, number of children, family size, supplement and medications use, physical activity, dietary intake of fiber and magnesium(OR: 0.83; 95% CI: 0.39-1.79, *P* = 0.65). Differences in obesity and overweight between tertiles of GI were also non-significant (OR: 0.75; 95% CI: 0.42-1.31, *P* = 0.31), even after adjustment for the potential confounders (OR: 1.54; 95% CI: 0.70-3.40, *P* = 0.26).

**Table 4:** Association of dietary glycemic index and glycemic load and the risk of overweight and obesity among Tehranian women.

	**Glycemic index**				**Glycemic load**			
	**1**	**2**	**3**	***P*** ^a^	**1**	**2**	**3**	***P***
Crude	1	0.87 (0.49,1.51)	0.75 (0.42,1.31)	0.31	1	1.08 (0.61,1.93)	1.80 (1.02,3.16)	0.03
Model 1^1^	1	0.89 (0.47,1.66)	1.03 (0.54,1.95)	0.92	1	0.94 (0.49,1.80)	1.79 (0.89,3.62)	0.11
Model 2^2^	1	0.83 (0.43,1.60)	0.86 (0.43,1.71)	0.67	1	0.62 (0.34,1.37)	1.48 (0.70,3.16)	0.35
Model 3^3^	1	0.81(0.40,1.64)	0.83 (0.39,1.79)	0.65	1	0.70 (0.35,1.41)	1.54 (0.70,3.40)	0.34

^a^ *P* values are from logistic regression. ^1^Model 1: Adjusted for age and energy intake. ^2^Model 2: Further adjusted for marriage status, education, occupation, number of children, family size, supplement use, medications use, and physical activity (METs/d). ^3^Model 3: Further adjusted for dietary intake of fiber and magnesium.

## Discussion

Findings from this cross-sectional study did not show significant association between dietary GI/GL and odds of high SBP/DBP. In addition, we could not find a significant association between dietary GI/GL and risk of obesity and overweight. Although there were differences of energy intake with GI and GL between individuals, data are adjusted for energy intake in Table 2.

These findings are in the same line with a study by Sloth et al in which differences in DBP and SBP were not significant in participants who consumed a low GI diet than those with a high GI diet.^21^ Moreover, another study in 2010 did not find a significant association between dietary GI/GL and SBP/DBP, among 878 postmenopausal women.^35^ On the other hand, some other studies have found a significant association between dietary GI/GL and BP in adults and aged peoples.^23,36-39^ It should be noted that participants in these studies were from both genders and suffered from hypertension. Therefore, one may expect that the association of dietary GI/GL with BP is different among males comparing to females.

It is necessary to mention consuming a high carbohydrates diet enhances postprandial glycemia and insulin secretion at various speeds, depending on the source of carbohydrates as well as amount and type of the dietary fibers.^40^ Therefore, quality and quantity of the ingested carbohydrates are the principal determinants of postprandial glycemic response.^41^

In this study, we also found that women who consumed a diet with higher GI were more likely to have higher intakes of vitamin B1, vitamin B2, vitamin C, β-carotene, calcium, and potassium. Also, there was a direct association between dietary GL and intakes of some nutrients including vitamin C, B1, B2, P, and calcium. Regarding to these findings, it can be suggested that those with the higher dietary GI/GL have also higher intakes of some vitamins and minerals which have found to have anti-hypertension effects.^42-44^

In current study, we found a direct association between dietary GL and odds of overweight and obesity, however, after adjustment for the potential confounders this association disappeared. In addition, we failed to find a significant association between dietary GI and odds of overweight and obesity, even after adjustment for the potential confounders. In line with our findings, some studies could not find differences in body weight between subjects who consumed a high or a low GI diet.^21,45,46^ In contrast, some other studies have reported an inverse association between consumption of a low GI diet and body weight.^47,48^ These different findings may be partially due to differences in study design, target population, and dietary assessment tools used to determine dietary GI and GL.

To the best of our knowledge, current study is the first study investigating association of dietary GI/GL with high BP among adult women in Iran. Some limitations should be kept in the mind. Due to the cross-sectional design of this study, causality could not be discovered. Hence, future researches, in particular randomized clinical trials are required to confirm these findings and to specify the causality. Although, we used a validated questionnaire to estimate dietary intakes of participants, FFQ has not been particularly planned to evaluate dietary GL and GI, therefore, it should be used carefully. Besides, due to using FFQ, misclassification of study participants is also probable.

In conclusion, we could not find significant association between dietary GI/GL and the risk of high SBP/DBP, even after controlling for a wide range of potential confounding factors. In addition, no association was found between dietary GI/GL and odds of overweight or obesity in adult women. Further studies, in particular large scale clinical trials are required to shed light in this area.

## Competing interests

The authors have no conflicts of interest to declare and they did not use any outside assistance in preparing the manuscript.

## Ethical approval

The study protocol was approved by the local ethics committee of the Tehran University of Medical Sciences (Code: BN092).

## Acknowledgments

This study was funded by the National Elites Foundation in Iran (Code: BN092) and Iran National Science Foundation and School of Nutritional Sciences and Dietetics, Tehran University of Medical Sciences (TUMS).
